# Dorsal Approach for Corticosteroid Injection in Trigger Finger Management: A Scoping Review

**DOI:** 10.1177/22925503251379892

**Published:** 2025-10-01

**Authors:** Muhammadhasan Nasser, Kaitlyn Soro, Natasha Barone, Kevin J. Zuo

**Affiliations:** 1 Temerty Faculty of Medicine, 12366 University of Toronto, Toronto, Canada; 2 Faculty of Health Sciences, 4257 Queen's University, Kingston, Canada; 3 Division of Plastic, Reconstructive & Aesthetic Surgery, Department of Surgery, 7938 University of Toronto, Toronto, Canada; 4 Division of Plastic & Reconstructive Surgery, Toronto Western Hospital, 7989 University Health Network, Toronto, Canada

**Keywords:** stenosing tenosynovitis, trigger finger, corticosteroid injection, efficacy, pain, ténosynovite sténosante, doigt à gâchette, injection de corticostéroïde, efficacité, douleur

## Abstract

**Introduction:** Stenosing tenosynovitis (trigger finger) is a common condition caused by inflammation and hypertrophy of flexor tendons. Corticosteroid injection (CSI) is an effective and safe treatment option. A palmar approach for CSI is typically used; however dorsal injection, which may be less painful, is not well-studied. **Methods:** A 6-stage scoping review was conducted to characterize outcomes associated with dorsal CSI for trigger finger management. We searched Ovid MEDLINE, EMBASE, and Web of Science for eligible articles in English from inception to July 2024. Data regarding study characteristics and CSI outcomes were synthesized. Risk of bias was assessed using Joanna Briggs Institute Critical Appraisal Tools. **Results:** Four articles were included in the review, comprising 1 case series, 2 cohort studies, and 1 randomized controlled trial (RCT). Symptom resolution rates following dorsal CSI ranged from 54% to 73.5%, comparable to a palmar approach. Two studies compared dorsal and palmar injections and found no significant differences in effectiveness. Pain scores for dorsal injections were similar or significantly lower than those for palmar injections in 2 studies. No adverse effects or complications were reported with either injection technique. **Conclusion:** Current evidence suggests a dorsal approach for CSI in trigger finger management is noninferior to a palmar approach in terms of efficacy and safety, with potential benefits in reducing injection-associated pain. However, more high-quality studies, including RCTs, are needed. Future research should assess anesthetic distribution and patient-reported outcomes to better understand the clinical implications of a dorsal CSI approach.

## Résumé

**Introduction :** La ténosynovite sténosante (doigt à gâchette) est un trouble fréquent provoqué par l’inflammation et l’hypertrophie des tendons fléchisseurs. L’injection de corticostéroïdes (ICS) est une option thérapeutique efficace et sûre. Habituellement, on utilise un abord palmaire pour l’ICS; néanmoins, l’injection dorsale qui peut être moins douloureuse, n’est pas bien étudiée. **Méthodes :** Une revue exploratoire en 6 étapes a été menée pour identifier les résultats associés à l’ICS par voie dorsale pour la gestion du doigt à gâchette. Nous avons recherché les articles en langue anglaise admissibles dans les bases de données Ovid, MEDLINE, EMBASE et Web of Sciences, depuis leur création jusqu’en juillet 2024. Les données portant sur les caractéristiques des études et l’ICS ont été synthétisées. Le risque de biais a été évalué en utilisant les outils d’évaluation critique (Critical Appraisal Tools) de l’Institut Joanna Briggs. **Résultats :** Quatre articles ont été inclus dans la revue, dont une série de cas, deux études de cohortes et un essai contrôlé randomisé (ECR). Les taux de résolution des symptômes après l’ICS par voie dorsale étaient compris entre 54% et 73,5%, comparables à ceux de l’abord palmaire. Deux études ont comparé les injections par voie dorsale et par voie palmaire et n’ont trouvé aucune différence significative sur le plan de l’efficacité. Les scores de douleur pour les injections par voie dorsale étaient similaires ou significativement inférieurs à ceux des injections palmaires dans les deux études. Aucun effet indésirable et aucune complication n’ont été signalés avec chacune des deux techniques. **Conclusion :** Les données probantes actuelles suggèrent qu’un abord dorsal pour l’ICS dans la gestion du doigt à gâchette n’est pas inférieur à l’abord palmaire sur les plans de l’efficacité et de la sécurité, et s’accompagne d’avantages potentiels en réduisant la douleur associée à l’injection. Cependant, des études de meilleure qualité, y compris des ECR, sont nécessaires. La recherche future devrait évaluer la répartition anesthésique et les résultats rapportés par les patients pour mieux comprendre les implications cliniques de l’ICS par abord dorsal.

## Introduction

Stenosing tenosynovitis (trigger finger) is one of the most common presentations seen by hand surgeons.^1,2^ The pathophysiology of trigger finger involves hypertrophy and inflammation of flexor tendon within its synovial sheath, causing friction and impingement with tendon gliding particularly at the A1 pulley.^3–6^ Patients with trigger finger present with pain and stiffness of the affected digit(s) and may also experience clicking or locking of their finger(s).^3,7^

Initial management of trigger finger is nonsurgical, involving rest, splinting, nonsteroidal anti-inflammatory drugs, or corticosteroid injection (CSI).^7,8^ CSI has a reported resolution rate between 45% and 87% with a single injection and an overall success rate of 47% to 92% after multiple injections.^5,9–13^

CSI for trigger finger is typically performed through the palm at the level of the A1 pulley of the affected digit.^5,12^ Various other methods of CSI have been described in the literature, including palmar injections more distally along the affected digit, or injecting through the dorsal aspect of the hand via the webspace. Very few studies have evaluated a dorsal approach to CSI for trigger finger despite a widespread though controversial notion that the dorsal skin of the hand is less sensitive—and thus less painful to inject—than the palmar surface of the hand.^14,15^ This belief influences regional anesthesia techniques, where a 2-injection dorsal digital block, rather than a single palmar injection, may be preferred by clinicians to minimize injection-associated pain.^15–17^ Other studies suggest that a single palmar injection may be equally or less painful for patients than a 2-injection dorsal digital block.^14–18^ Notably, CSI for trigger finger involves only a single injection, regardless of whether it is administered from the palmar or dorsal aspect of the hand.

Given the lack of clarity, this scoping review aims to summarize the literature on dorsal webspace CSIs for patients with trigger finger and describe associated outcomes; specifically, injection-associated pain, distribution of anesthesia, effectiveness, and adverse effects. These findings can inform practice recommendations on CSI techniques for trigger finger and are crucial in evaluating the potential of this technique to improve the needle experience and reduce pain for patients.

## Methods

This scoping review adheres to the 6 stages as outlined by Arksey and O’Malley^19^ and further expounded on by Levac and colleagues.^20^ The Preferred Reporting Items for Systematic reviews and Meta-Analyses extension for Scoping Reviews (PRISMA-ScR) Checklist^21^ was also used in the development and reporting of this scoping review.

### Stage 1: Identifying the Research Question

This scoping review seeks to understand what is known from the existing literature about the effectiveness, pain, and safety of a dorsal approach for CSI in trigger finger management.

### Stage 2: Identifying Relevant Studies

The search strategy focused on terms related to trigger finger, steroids, injections, and pain outcomes (Table 1). The searches were conducted in Ovid MEDLINE, Web of Science, and EMBASE in July 2024.

**Table 1.** Search Strategies and Databases.

Databases	Search terms
Ovid MEDLINE	(“Trigger Finger Disorder” [MeSH] OR “stenosing tenosynovitis” [keyword] OR “trigger finger” [keyword] OR “trigger digit” [keyword]) AND (“steroids” [MeSH] OR “Adrenal Cortex Hormones” [MeSH] OR “steroid” [keyword] OR “corticosteroid” [keyword]) AND (“injections” [MeSH] OR “Anesthesia, Local” [MeSH] OR “injection” [keyword]) AND (“dorsal” [keyword] OR “webspace” [keyword] OR “Fingers” [MeSH]) AND (“Pain” [MeSH] OR “pain” [keyword] OR “sens*” [keyword] OR “Analgesia” [MeSH] OR “analges*” [keyword] OR “Anesthesia” [MeSH] OR “anesthe*” [keyword] OR “Nociception” [MeSH] OR “nocicepti*” [keyword])
Web of Science, EMBASE	(stenosing tenosynovitis OR trigger finger OR trigger digit) AND (steroid OR corticosteroid) AND (injection OR local) AND (dorsal OR webspace) AND (pain OR sens* OR analges* OR anesthe* OR nocicept*)

Literature management software (Covidence) was used for reference management, duplicate removal, title and abstract screening, full-text review, and data extraction.

### Stage 3: Study Selection

The inclusion and exclusion criteria for selecting studies to include in this review (Table 2) were developed *posthoc* as we gained familiarity with the existing literature.^19^ After removing duplicate studies in Covidence, inclusion and exclusion criteria were applied in a 2-stage screening process. First, 2 independent reviewers screened titles and abstracts to identify relevant literature. Any discrepancies in screening were resolved collaboratively after open discussion. Thereafter, the same 2 authors independently engaged in full-text review and any differences in inclusion were similarly resolved. Specific reasons for exclusion were recorded at the full-text review stage.

**Table 2.** Inclusion and Exclusion Criteria.

	Inclusion criteria	Exclusion criteria
Population	Age 18 years or older Diagnosis of stenosing tenosynovitis (trigger finger) of any of the 5 digits of hand	Age less than 18 years No diagnosis or diagnosis other than stenosing tenosynovitis (trigger finger)
Intervention	Digital corticosteroid injection (CSI) for trigger finger Dorsal approach	Do not receive CSI CSI injected into site other than the digit (eg, wrist CSI) Only palmar approach Surgical intervention (percutaneous or open)
Outcomes	Pain Sensation (if combined local anesthesia) Symptom resolution Need for repeat injection Side effects Adverse outcomes	
Study characteristics	Original research (eg, case report, case series, observational studies, controlled trials) Published during or after 1931	Not in English (or untranslated) Literature reviews (eg, systematic reviews, scoping review, narrative reviews) Commentaries or editorials Gray literature (ie, nonpeer-reviewed literature) Nonclinical or nonhuman studies (eg, anatomic/cadaveric studies, animal models) Published before 1931

Overall, we included original clinical studies with adult patients diagnosed with trigger finger who received a CSI via a dorsal injection technique. Outcomes included pain, anesthesia (if applicable), efficacy, and adverse outcomes or complications. Studies were excluded if they involved pediatric patients, patients without a diagnosis of trigger finger, patients who received management other than a CSI (eg, A1 pulley release), or if the studies were not in English (or not translated to English).

### Stage 4: Charting the Data

A data extraction form was developed, tested, and revised by 2 members of the research team who also completed extraction. After independent extraction was complete, discrepancies in extracted data were collectively reviewed and finalized based on consensus.

Effectiveness of dorsal CSI was defined as either complete symptom resolution or the proportion of patients not requiring a repeat injection for trigger finger symptoms. Pain was defined as measured using a validated pain score, such as the visual analogue scale (VAS) or numerical rating scale. Specifically, the VAS for pain is typically scored out of 10, with higher scores indicating more pain.^22^

#### Assessment of Risk of Bias

Based on recommendations by Levac and colleagues,^20^ we also conducted a methodological quality assessment using the Joanna Briggs Institute (JBI) Critical Appraisal Tools for case series, cohort studies, and randomized controlled trials (RCTs).^23–25^ Two reviewers discussed the JBI Critical Appraisal Tools to establish shared understanding before independently critically appraising each included article using the appropriate JBI tool and then reviewing appraisals collectively for agreement.

In order to summarize the risk of bias for the included studies, we classified the studies based on the proportion of JBI checklist items answered as “Yes.” Specifically, we followed the rating score system as described by Kanda and colleagues^26^: very poor quality (0%-25% “Yes”); poor quality (26%-50% “Yes”); fair quality (51%-75% “Yes”); good quality (76%-100% “Yes”).

### Stage 5: Collate, Summarize, and Report the Results

The following data were extracted and reported from included studies: study characteristics, setting, study population, intervention characteristics, outcomes (specifically measures of pain, sensation, effectiveness, and complications), and main findings.

### Stage 6: Consultation Exercise

As recommended by Arksey and O’Malley^19^ and re-emphasized by Levac and colleagues^20^ we engaged in consultation with an academic hand surgeon and junior plastic surgery resident familiar with trigger finger management, specifically dorsal CSI techniques. After completing Stage 5 of the scoping review, both clinicians critiqued the findings of the review, provided content expertise to contextualize the meaning of the results, and highlighted areas for potential further investigation.

## Results

**Figure 1.**
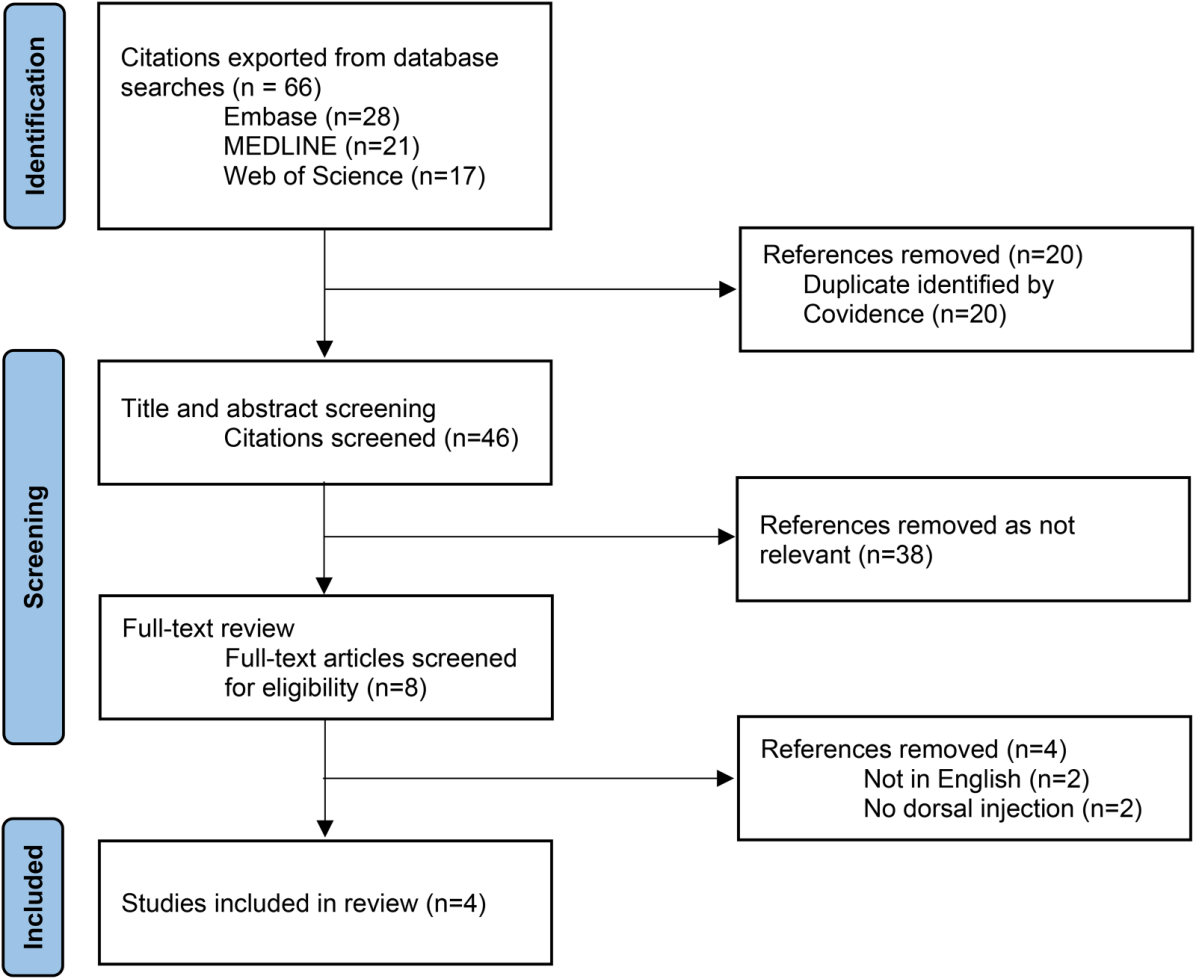
PRISMA flow chart of literature inclusion and exclusion process.

Searches of the databases were completed in June 2024 and yielded 66 articles. After the removal of duplicates, title and abstract screening, and full-text review, 4 articles were included (Figure 1).

PRISMA, Preferred Reporting Items for Systematic reviews and Meta-Analyses.

### Study Characteristics and Setting

Included studies consisted of a case series (*n* = 1), cohort studies (*n* = 2), and an RCT (*n* = 1). Geographically, studies took place in Spain (*n* = 2), France (*n* = 1), and the United States (*n* = 1). All participants were recruited in surgeons’ clinics.

### Study Population

All studies included adult patients with a diagnosis of trigger finger. Exclusion criteria were reported by 3 studies which included, omitting patients with arthritis, prior CSI, and/or surgical treatment of the affected digit(s).^27–29^ Rosenbaum and colleagues also excluded all patients with diabetes mellitus (Tables 3 and 4).^29^

**Table 3.** Study Characteristics and Population.

Study	Location	Funding	Design	Inclusion criteria	Exclusion criteria	Duration of follow-up
Buch-Jaeger et al, 1992	France	Not reported	Case series	Diagnosis of trigger finger	Not reported	337 days
Rosenbaum et al, 2018	United States	None	Prospective cohort	Age ≥18 years Single idiopathic trigger digit (Patel and Moradia Grade 1 to 3) No prior injection into affected digit Understands English Symptoms <12 months	Comorbid endocrine disorders Rheumatoid arthritis Receiving renal dialysis Age <18 years Locked trigger digit Symptoms >12 months	8 weeks
Jiménez et al, 2020	Spain	Foundation of the Colegio Oficial de Médicos de Las Palmas	Randomized controlled trial	Adults (age ≥18 years) Diagnosis of trigger finger	Allergy to injection ingredients Uncontrolled diabetes Uncontrolled hypertension Inflammatory or autoimmune arthritis Previous injection or surgery of the involved digit Multiple trigger fingers Suspected pregnancy or breastfeeding	12 months
Jiménez et al, 2022	Spain	Foundation of the Colegio Oficial de Médicos de Las Palmas	Prospective cohort	Adults (age ≥18 years) Diagnosis of trigger finger	>1 trigger finger Allergy to injection ingredients Uncontrolled diabetes (HbA1c >8%) Inflammatory/autoimmune arthritis Previous injection or surgery to affected finger Pregnancy or breastfeeding	12 months

**Table 4.** Study Interventions and Demographics.

Study	Interventions	Steroid, dose	Local anesthetic, dose	Needle (gauge)	Infiltration	Sample size	Age in years (mean, SD)	Sex (% female)	Diabetes (% Yes)
Buch-Jaeger et al, 1992	Dorsal injection	Hydrocortisone	Lidocaine, 1%	25	Transthecal	210 digits	55	71	10
Rosenbaum et al, 2018	Dorsal webspace injection	Dexamethasone, 4 mg	None	27	Transthecal	5 digits	58.8, 5.4	40	n/a
	Proximal palmar injection	Dexamethasone, 4 mg	None	27	Transthecal	22 digits	54.7, 10.2	63.6	n/a
	Proximal phalanx injection	Dexamethasone, 4 mg	None	27	Transthecal	12 digits	62.5, 6.5	50	n/a
	Overall					39 digits	57.7, 9.3	56.4	n/a
Jiménez et al, 2020	Dorsal webspace injection	Betamethasone, 6 mg	Mepivicaine, 2%	25	Subcutaneous	84 patients	61	71	12
	Palmar midline injection	Betamethasone, 6 mg	Mepivicaine, 2%	25	Subcutaneous	76 patients	59	74	8
	Overall					160 patients	60	72.5	10
Jiménez et al, 2022	Dorsal webspace injection	Betamethasone, 6 mg	Mepivicaine, 2%	25	Subcutaneous	126 patients	61	68	11

### Intervention Characteristics

All studies (*n* = 4) involved CSI for trigger finger delivered through the dorsal aspect of the hand via the webspace. Two studies compared a dorsal webspace CSI to 1 or more palmar CSI techniques: proximally over the metacarpal head at the A1 pulley (*n* = 2)^27,29^ or distally in the palmar surface of the first phalanx of the affected digit (*n* = 1).^29^ Half the studies (*n* = 2) employed subcutaneous injection^27,28^ and the other half (*n* = 2) used a transthecal injection technique.^29,30^

Three different steroids were used in the CSI across the 4 studies; specifically, betamethasone,^27,28^ dexamethasone,^29^ or hydrocortisone.^30^ Most studies (*n* = 3) used a combined local anesthetic: mepivacaine 2%^27,28^ or lidocaine 1%.^30^

### Outcomes

#### Effectiveness

All studies included reported on CSI effectiveness, either as symptom resolution and/or reinjection rates for symptomatic persistence or recurrence.

Buch-Jaeger and colleagues^30^ reported a 73.5% (128 of 174) symptom resolution rate 1 month after a single CSI. Of those with complete symptom resolution at 1 month, 82% (98 of 119) had no symptom recurrence at 6 months post-CSI. At 12 months, 21% (25 of 119) of all patients followed required a second CSI.

Rosenbaum and colleagues^29^ found that 40% (2 of 5) of patients receiving a dorsal webspace CSI required repeat injection at 4 or 8 weeks, compared to 66.7% (8 of 12) in both the proximal palmar and proximal phalanx groups. Overall, however, the authors found no statistically significant difference in repeat injection rates across the 3 different injection sites.

In their RCT, Jiménez and colleagues^27^ found that the initial CSI was successful in 51% (37 of 72) of patients in the dorsal webspace arm compared to 47% (31 of 66) in the palmar arm; after repeat injections, the overall symptom resolution rates at 12 months were 67% (48 of 72) for the dorsal technique and 56% (37 of 66) in the palmar technique. At 3 months postinitial CSI, there were an equal number of recurrences (*n* = 4) in each group. None of the abovementioned results achieved statistical significance.

Jiménez and colleagues^28^ reported a 54% (62 of 114) symptom resolution rate at 12 months for a single dorsal injection and 66% (16 of 24) for a second CSI. The overall success rate was 68% (78 of 114). There were 4 symptomatic recurrences at 12 months of follow-up.

#### Sensation

None of the studies that used a combined local anesthetic in the injection (*n* = 3) assessed or reported sensation or anesthetic distribution for dorsal CSI.^27,28,30^

#### Pain

Three of the 4 studies included in this review reported injection-associated pain using the VAS.

Rosenbaum et al^29^ found that dorsal CSI were associated with a 6.8 (1.8) mean (SD) VAS score, compared to 6.6 (2.6) for proximal palmar injection and 6.0 (2.8) for proximal phalanx injection, but no statistical significance (*P* = .754) in pain scores overall.

Jiménez and colleagues^27^ reported a statistically significant difference in mean (SD) VAS pain scores; 3.6 (2.4) for dorsal injection and 5.4 (2.7) for palmar midline injection (*P* < .001).

Jiménez et al^28^ reported an overall mean (SD) VAS score of 3.8 (2.3) for dorsal CSI.

#### Complications

None of the 4 included studies reported any adverse effects or complications associated with dorsal CSI for trigger finger. No complications were reported for any palmar injection techniques also evaluated in the included studies (Table 5).^27–30^

**Table 5.** Study Results by Intervention.

Study	Interventions	Complete symptom resolution after first CSI (%)	Overall symptom resolution (%)	Reinjection rate (%)	VAS pain (mean, SD)
Buch-Jaeger et al, 1992	Dorsal injection	73.5	82.8	21	Not reported
Rosenbaum et al, 2018	Dorsal injection	0	Not reported	40	6.8 ± 1.8
	Proximal palmar injection	0	Not reported	66.7	6.6 ± 2.6
	Proximal phalanx injection	8.3	Not reported	66.7	6.0 ± 2.8
	Overall	2.6	Not reported	46.2	6.5 ± 2.5
Jiménez et al, 2020	Dorsal webspace injection	51	67	Not reported	3.6 ± 2.4
	Palmar midline injection	47	56	Not reported	5.4 ± 2.7
	Overall	49.3	61.6	18.8	Not reported
Jiménez et al, 2022	Dorsal webspace injection	54	68	21	3.8 ± 2.3

Abbreviations: CSI, corticosteroid injection; VAS, visual analogue scale.

#### Quality Assessment

Of the 4 included studies, 2 were of good quality and 2 of fair quality. The case series^30^ lacked clear inclusion/exclusion criteria and did not describe how they identified or measured trigger finger in participants. The RCT did not conceal allocation and no blinding was used.^27^ Both observational studies did not describe strategies to address incomplete follow-up (Table 6).^28,29^

**Table 6.** Joanna Briggs Institute Quality Assessment.^23–30^

Study	Items selected “Yes” (%)	Quality assessment
Buch-Jaeger et al, 1992	7 of 10 (70%)	Fair quality
Rosenbaum et al, 2018	10 of 11 (91%)	Good quality
Jiménez et al, 2020	8 of 12 (67%)	Fair quality
Jiménez et al, 2022	10 of 11 (91%)	Good quality

## Discussion

This scoping review examined the available evidence on the use of dorsal CSI for managing trigger finger. Despite the prevalence of trigger finger and the frequent use of CSI as a nonoperative treatment, there is limited evidence evaluating a dorsal approach for CSI. Furthermore, only 2 of the 4 studies compared dorsal and palmar injection techniques for trigger finger management, and we identified only 1 RCT comparing dorsal and palmar injections, highlighting the need for more high-quality studies on this topic.

### Effectiveness

Symptom resolution with dorsal CSI was similar to rates reported in the literature. Both studies which compared dorsal and palmar injection techniques found similar or slightly better symptom resolution and reinjection rates for dorsal CSI but no statistically significant differences. Overall, this review suggests that a dorsal approach for CSI is noninferior to palmar injection for managing trigger finger.

### Sensation

None of the studies which used a combined anesthetic agent assessed the distribution of anesthesia or changes in sensation postinjection. Future research should address this gap and compare the anesthetic distribution in both palmar and dorsal CSI as this has the potential to influence postinjection pain experiences for patients with trigger finger.^31^

### Pain

Pain associated with CSI for trigger finger is not insignificant for patients.^31,32^ This review found that VAS scores for pain associated with dorsal CSI were consistent with or lower than scores reported in the literature for palmar CSI.^31^ When compared to palmar injection techniques, VAS pain scores for dorsal CSI were similar^29^ or significantly lower,^27^ suggesting there may be a potential advantage to a dorsal approach in reducing patient discomfort during injection. It is important to note that VAS pain scores were comparatively higher for all 3 injection techniques evaluated by Rosenbaum and colleagues^29^ which may be due to the lack of a combined local anesthetic, impacting the pain scores reported soon after injection.

### Complications

None of the studies in this review reported any adverse events or complications from dorsal CSI, suggesting it may be a similarly safe alternative to palmar CSI in trigger finger management.

### Quality Assessment

In the 4 studies included in this review, we determined 2 studies were of good quality, while the remaining 2 were of fair quality. Notably, there was an absence of allocation concealment and blinding in the RCT^27^; however, one must consider the practical difficulty in blinding for a study comparing injection locations and techniques, especially where a main outcome is pain associated with the intervention itself. Nevertheless, this review highlights the need for more rigorously designed and reported studies to better evaluate the effectiveness and safety of dorsal CSI for trigger finger.

### Limitations

This review has some limitations. Most studies included (*n* = 3) were case series or observational studies, and only half of them (*n* = 2) compared dorsal CSI to palmar techniques. We recommend researchers conduct more high-quality trials directly comparing outcomes for palmar and dorsal CSI in trigger finger management. Additionally, the plane of CSI differed among studies, however, this is unlikely to substantially influence the results as transthecal and subcutaneous CSI have been found to have generally similar outcomes in trigger finger management.^33^ Our review may also be impacted by publication bias; specifically, studies that may have found dorsal CSI to have worse or equivocal outcomes in trigger finger treatment may not have been submitted or accepted for publication, skewing the available evidence.^34^

### Implications for Practice

When choosing an approach for CSI in trigger finger, clinicians might consider medical evidence, patient-specific factors, and personal familiarity with injection techniques. This review revealed that, while current evidence does not definitively favor a dorsal approach over a palmar approach for CSI in trigger finger management, the potential for reduced injection-associated pain with the dorsal approach in combination with evidence of similar effectiveness and safety warrants further consideration.

### Future Directions and Recommendations

Given the sparse evidence on dorsal CSI in the context of trigger finger, particularly the limited number of controlled studies, future research should focus on well-designed RCTs with larger sample sizes and standardized outcome measures in order to provide more robust evidence as to the optimal CSI technique for trigger finger. These studies should aim to directly compare dorsal and palmar CSI techniques, including assessments of pain, anesthetic distribution (where applicable), symptom resolution, and adverse effects. Additionally, researchers should consider qualitative or mixed-method studies characterizing clinicians’ awareness, familiarity, and comfort with dorsal CSI techniques for managing trigger finger.

## Conclusion

This scoping review found that a dorsal CSI for trigger finger may offer comparable effectiveness and safety with potentially lower injection-associated pain compared to a palmar approach; however, the current literature is insufficient to make conclusive clinical recommendations. Future research should address existing gaps and methodological limitations to better guide clinical decision-making and improve patient experiences when performing CSI for managing trigger finger.

## Supplemental Material

sj-docx-1-psg-10.1177_22925503251379892 - Supplemental material for Dorsal Approach for Corticosteroid Injection in Trigger Finger Management: A Scoping Review Supplemental material, sj-docx-1-psg-10.1177_22925503251379892 for Dorsal Approach for Corticosteroid Injection in Trigger Finger Management: A Scoping Review by Muhammadhasan Nasser, Kaitlyn Soro, Natasha Barone and Kevin J. Zuo in Plastic Surgery
